# A study evaluation framework for measuring cognition. Lessons learned in cross-national contexts from four English-speaking aging cohorts

**DOI:** 10.21203/rs.3.rs-5574616/v1

**Published:** 2024-12-20

**Authors:** Shabina Hayat, Sarah Assaad, Carol Brayne, Nasrin Ahmed, Andrew Steptoe

**Affiliations:** University College London; University College London; University of Cambridge; Anglia Ruskin University - Chelmsford Campus: Anglia Ruskin University; UCL: University College London

## Abstract

The Harmonized Cognitive Assessment Protocol (HCAP) is a detailed battery assessing cognition among older people used by studies across the world. Data harmonization is a key priority for HCAP studies. We used a mixed-methods approach using established theories from the existing literature detailing the methodologies of longitudinal studies and from the implementation of HCAP in four English-speaking studies adopting the same protocol. Through a detailed investigation involving the English Longitudinal Study of Ageing (ELSA), the Health and Retirement Study (HRS), The Irish Longitudinal Study on Ageing (TILDA), and the Northern Ireland Cohort for the Longitudinal Study of Ageing (NICOLA), we identified 60 factors contributing to the development of a conceptual framework for the evaluation and implementation of HCAP. We present this framework and a prototype checklist as a tool for providing a transparent and structured approach to improve data quality, cross-country comparability and for identifying, mitigating, and monitoring sources of bias. The framework consisting of four broad headings: (1) Organisation and design, (2) Competency of personnel and systems, (3) Implementation and outputs, and (4) Feedback and communication. Studies seeking to harmonize results in cross-national contexts should give operational aspects of fieldwork careful consideration as part of the harmonization process.

## Introduction

Observational studies are currently a major source of evidence on risk factors and potential causes of dementia. [1–3] It is becoming increasingly clear that these real-world population-based studies need to work towards presenting robust evidence for inference of causality rather than reporting on associations alone to better inform effective prevention and treatment. [4] Furthermore, there is a push to combine evidence from diverse settings. [4] Observational studies of aging face methodological challenges [2, 5] as they attempt to isolate or tease out individual risk factors that are inextricably interconnected and change over time. It is difficult to disentangle whether an observed difference is a true difference, residual confounding or as an artefact due to the variability in methodology and design. There is a concerted effort amongst dementia researchers to develop robust analytical approaches and share “best practices.” [6–10] Most of this effort focuses on more generic aspect of methodology and study design of longitudinal studies of aging, with very little published on the operational functions and the management and monitoring of fieldwork.

The Health and Retirement Study (HRS) initiated in 1992, is a longitudinal study investigating health, economics, and demographics of aging and the retirement process in aging adults in the United States. [11] HRS has been used as a template for a growing network of international aging studies (https://hrs.isr.umich.edu/about/international-family-studies) sharing common scientific goals with a core function to harmonize data to allow for cross-study comparisons of their aging populations. The Harmonized Cognitive Assessment Protocol (HCAP), a comprehensive battery of established neuropsychological assessments along with informant (family or friend) report to measure cognitive function was developed by HRS and implemented to a sub-sample of their cohort aged 65 years and above in 2016. [12] HCAP has since been administered in a range of other studies across the world. (https://hcap.isr.umich.edu/)

Although HCAP was designed to allow for comparability across countries, modifications have been necessary to suit the language, culture, education and socio-economic features of the target population to allow a better fit within local contexts. [13–16]. Data harmonization across different countries and settings is challenging and requires careful consideration of cross study variations to allow for meaningful comparisons. [4, 17] The importance pre-statistical harmonization, which is a qualitative process to determine equivalence of variables and consistency of cognitive data applied prior to any statistical implementation has been reported. [18–22] Despite the immense efforts for harmonization of methods for the established cognitive and neuropsychological assessments in the HCAP network which included review of study design, administration and scoring of cognitive tests, [21–23] impact of the operational aspects in the implementation HCAP have not been explored.

In this study we aim to explore and compare differences in administration, variability in scoring as well as in management and monitoring of fieldwork in four English-speaking HCAP cohorts. We will use these findings to develop a framework as a tool for study evaluation and implementation of HCAP and offer recommendations. This study seeks to inform fieldwork practices to improve the quality of data and cross-country comparability, contributing to the efforts of pre-statistical harmonization of the HCAP battery across diverse populations. The findings have broader reach, however, and will be of value to all those seeking to harmonize results across studies or implement primary fieldwork across settings.

## Methods

### Study Samples

Four English-speaking studies from the HCAP network were included in this study. (Table 1) Details of the baseline studies are reported elsewhere. [11, 12, 24–26] All four studies followed the same protocol for the implementation of HCAP with minor adjustments to allow for the local setting.

### Study Design

This study employed a mixed-methods design. Firstly, the authors used a deductive approach synthesising evidence from the existing literature to identify themes and categories to inform an online questionnaire and semi-structured interview. Secondly, through analysis of the data generated through the questionnaire and interviews, in addition to Public and Participant Involvement (PPI) and Focus Group activities, inductive analysis was employed to identify key themes for the creation and development of the conceptual framework for evaluation and implementation of fieldwork and operationalisation of HCAP.

### Creating the conceptual Framework

**Figure 1:**
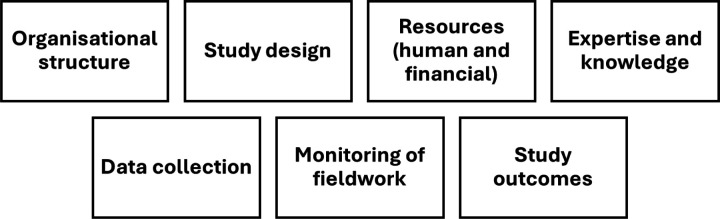
A priori conceptual framework for evaluation and implementation of fieldwork and operationalisation of HCAP

The first step was to construct an *a priori* framework based on experiences of the two authors (SH/SA) who had previously worked with longitudinal aging studies [27–29] (Figure 1). This framework was used as the foundation to direct the fieldwork and analysis.

#### Previous Research

We conducted a critical review of the literature, [30] using a systematic approach with explicit details of the search, synthesis and analysis. Medline, Google Scholar and PubMed were used to search for literature on methodologies and data collection in longitudinal studies. Articles in English language published from 1990 until 7th September 2023 were included. The purpose was to capture key themes with respect to training, monitoring, data capture, scoring, data accessibility and quality controlprocedures. Further hand searches were conducted by checking reference lists of relevant articles.

#### Deductive Analysis

The literature review was used to provide context and explore whether the evidence base was consistent with the a priori conceptual framework. We searched the literature describing the data collection phase of cohort studies, searching specifically for key factors pertaining to implementing and evaluating fieldwork. Using a deductive approach, we identified themes and subthemes from the literature that allowed deeper understanding of fieldwork implementation management and monitoring and tried to operationalise these constructs into question items that could be measured. Details and a narrative synthesis of the critical literature review are given elsewhere. [31] The data were categorised into six themes giving the final headings for the online questionnaire (available on request). The headings for the questionnaire were as follows:

- Organisational structure
- Recruitment and training of fieldwork team
- Recruitment of participants
- Fieldwork management
- Monitoring (quality control)
- Data Collection (data capture, coding, and cleaning)

#### Data Collection (Online Questionnaire)

The online questionnaire was completed by senior personnel (stakeholders/study partners) involved in the operations and fieldwork for HCAP at the four studies. The questionnaire grouped related concepts under the same theme to allow a logical flow. This included communication, training, monitoring, data capture, scoring, data accessibility and quality control procedures. Depending on the nature of the factor being examined, questions varied in format including Likert scales, multiple-choice questions and open-ended questions.

The questionnaire included capture of case numbers to explore response rates, organisational/workforce structure and size and the time taken on the data collection phase. Each section of the questionnaire was designed to take no more than 20 minutes to complete. The decision on who completed the questionnaire was left to the key contact, and was open to principal investigators, data managers, fieldwork researchers, and interviewers. A section was completed by one person (the same person was able to complete more than one section, if applicable). Study partners were asked to provide data on the most recent HCAP wave only. ELSA and HRS had completed the second wave of data collection whereas NICOLA and TILDA had just completed the first.

#### Qualitative Interviews

Study partners who completed the online questionnaire were then invited for a semi-structured interview based on their responses on the questionnaire. Where responses from the questionnaire provided new insights or needed further exploration, additional lines of enquiry were created for the qualitative interviews. Methods of operationalisation of HCAP were compared across the four studies ELSA, HRS, NICOLA and TILDA. Each interview lasted one hour. (Further details on the summaries from the online questionnaire and interviews are given in Supplementary Information A)

##### Focus Group Discussions

To gain further understanding into the fieldwork, a focus group with 5 Interviewers from the ELSA-HCAP team was also conducted. Interviewers were asked to provide insights into the challenges they experienced in implementing ELSA-HCAP. (Detailed summaries given in Supplementary Information B)

#### Public and Participant Involvement (PPI)

In recent years, there has been a growing recognition of the importance of involving the public and patients/participants directly in the research process. [32] Two of the four of our studies reported having a PPI component in their research. NICOLA reported an advisory group that advised on the study design and participated in the HCAP pilot study and TILDA had a PPI group that met regularly. We posed the question to the TILDA PPI group as to what motivates or deters people from participating in studies that include a cognitive assessment. We also conducted a detailed interview with the PPI manager linked to the academic institution housing ELSA on the benefits of including PPI in a study. Also discussed were the challenges and barriers for older people taking part in cohort studies, particularly from ethnic minority communities. (Details given in Supplementary Information C) Findings from these PPI activities were used to identify additional themes from the participant’s perspective that may impact implementation to further develop the framework.

#### Inductive Analysis

**Figure 2:**
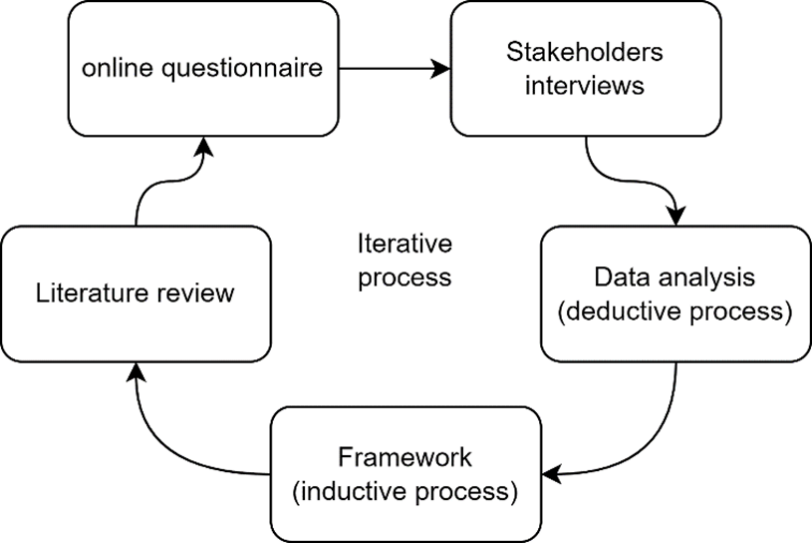
Flow diagram of iterative process for the development of themes and categories of the framework.

Data from the online questionnaire and the qualitative interviews were cleaned and organised. Due to the small number of participants and the straightforward organisation of the data, the authors used a manual approach using spreadsheet to organise and analyse the data. Engaging in a reflexive iterative process (Figure 2), authors (SH and SA) used both deductive (using prior knowledge and the literature review) and inductive analyses (using the data from the online questionnaire, qualitative interviews, the focus group and PPI activities to identify any new patterns and themes) to further build the framework.

**Figure 3:**
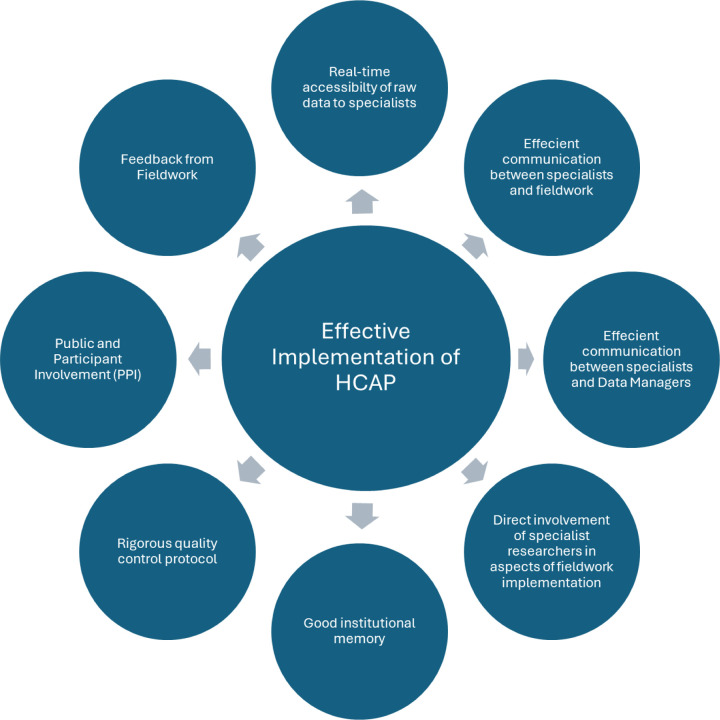
Diagrammatic representation of eight key facilitators of successful implementation of HCAP emerging from the Inductive analyses of online questionnaire, focus group and Public and Participant Involvement (PPI) activity.

The inductive analysis also revealed a total of eight elements that were identified as ‘key facilitators’ which had impact on either on the effective running of the study or data quality These are shown in figure 3. We gave a performance rating based how each ‘facilitator’ was implemented in the studies. This analysis further informed the development of our final framework. Not all factors were relevant in all four studies and so were coded as ‘Not applicable’. The findings of this analysis are shown in Supplementary Information D, Table S4).

## Results

### Fieldwork timeframes and study size

Table 2 shows a summary of the data collection time and size of the four studies. The largest study was HRS, followed by ELSA, TILDA and finally NICOLA. The time taken for fieldwork for the four studies varied from 6 months to a maximum of 24 months. ELSA and HRS employed lay interviewers whereas NICOLA and TILDA employed nurses and research assistants. ELSA had the largest number of interviewers (n=88) resulting in a much faster completion time of 6 months compared to over a year to 2 years for the other studies. HRS had 49 interviewers and NICOLA and TILDA had smaller data collection team of 6 interviewers each. Staff within NICOLA and TILDA had greater exposure and experience due to having conducted more interviews than those in ELSA and HRS.

### Recruitment of participants and response rates

The four studies varied in their methods of recruitment in terms of prioritising and targeting certain groups. ELSA and HRS included all participants who took part in the first HCAP wave. Only NICOLA targeted participants to include those with lower cognition. All four gave study participants financial incentives with HRS giving the highest amount. HRS and TILDA had the highest participant response rates (74%), followed by ELSA (61%) and then finally NICOLA with the lowest (42%). It is important to note that response rates are based on the numbers from the last interview stage of the core study from which the HCAP sample was drawn. All studies showed evidence of good communication, highlighting the importance of continued participation to the interviewers to promote positive experience for participants.

### Constructing the framework

**Figure 4:**
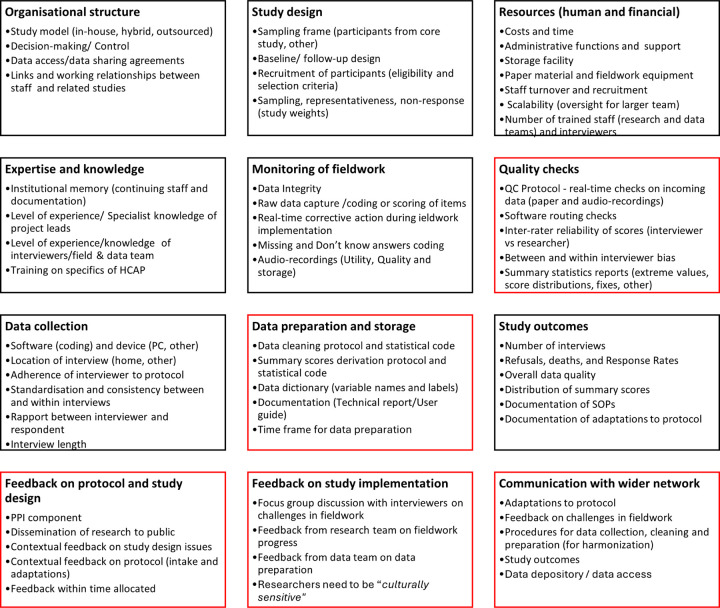
Visual representation of the proposed framework for the evaluation of HCAP study implementation and data quality.

In the final stage of the analysis, the data revealed a total of 60 factors that emerges as relevant to fieldwork implementation and data quality, not all which could be mapped into the categories of the original a priori framework (Figure 1). We restructured our framework to create a more coherent and organised structure that accurately reflected the essence of our analyses. Figure 4 is a visual representation of the final proposed framework consisting of four broad headings: (1) Organisation and design, (2) Competency of personnel and systems, (3) Implementation and outputs, and (4) Feedback and communication, with each heading further encompassing 3 themes each. The additional themes resulting from the analyses that were not a part of the original a priori conceptual framework are highlighted in red in the figure.

## Discussion

Using a mixed-methods design which included information from the methodology of longitudinal studies reported in the existing literature of aging and an in-depth analysis of the implementation and operationalisation of HCAP in the four English-speaking HCAP studies, we present a conceptual framework for study evaluation, planning, and implementation of HCAP for future studies.

Variation in the implementation, management, and monitoring (quality control) of a protocol in a cohort study of aging can have profound effects on the quality of data, leading to biased results, reduced validity, and compromising comparability across studies. Addressing these variations is critical, especially when the goal is to use the data for cross-study comparisons. Our results provide evidence that even when ‘adopting’ the same protocol and administering in the same language, there can be significant differences in the implementation, management and monitoring of HCAP across studies.

Response rate is considered to be an important metric of data quality and is used as an indicator of the representativeness of the populations from which they were sampled. We noted, as shown previously [33] that fieldwork efforts did have a positive influence on lower non-response across studies. Overall, response rates were expected to be higher for all four HCAP studies because participants were drawn from the latest wave of the core study. Participants who remain in the study at the last interview stage are more likely to be more engaged, committed, and compliant. NICOLA had the largest delay since the last wave as compared to the other studies (up to 5 years for some participants) and this may be part of the reason why NICOLA showed substantially lower response rates than the other studies. The study did identify other potential factors for lower response such as hesitancy by participants due to Covid and loss of contact. It was also the only study to target those with lower cognition in the core study. Further research is needed to understand the factors underlying variations observed in response rates as this was beyond the scope of this study and was not explored in detail. It is important for studies to ensure that those remaining in the sample still represent the population of interest and be aware of what biases might need to be considered.

Our findings have led to the creation of a framework that is empirically grounded. By including the discussions with the PPI manager and the PPI group, we were also able to include the lived experience and perspective of older people as an additional factor in our framework. The resulting framework consists of 60 factors, that sit within four broad headings: (1) Organisation and design, (2) Competency of personnel and systems, (3) Implementation and outputs, and (4) Feedback and communication, with each heading encompassing 3 themes each. We have translated this framework into a user-friendly checklist (*Supplementary Information E*) that efficiently highlights the key areas and propose this framework and the checklist as tools for evaluating and assisting current and future studies in the implementation of HCAP, and other similar studies.

### Organisation and design

The three themes mapped under this heading are (1) Organisational structure, (2) Study Design and (3) Resources. One key finding here was the different models of the organisational structure within the 4 studies, ranging from the in-house model (NICOLA and TILDA) where there was full integration of the specialist’ role with the fieldwork coordinating team, both situated within one organisation. The second was a hybrid model where the fieldwork team sits within a different group, although still under the same organisation (HRS) and the third was an outsourced model (ELSA), where the fieldwork was conducted in a different organisation to the specialist research team. This final model added a layer of complexity to fieldwork monitoring as well as data access. All 3 models were shown to work effectively with different strengths. Within the most complex outsourced model, what was shown to be paramount was good communication, effective working relationships, interpersonal connection and accessibility between the fieldwork team and the specialists. Another factor that was shown to be of considerable relevance was ‘institutional memory’, not only through documentation but also amongst the team overseeing fieldwork and the interviewers. Having the experience and insight of the wider study results in a knowledgeable and highly motivated research team.

### Competencies of personnel and systems

This heading includes (1) Expertise and knowledge, (2) Monitoring of fieldwork and (3) Quality checks. The type of personnel (Interviewers vs nurses or research assistants) did not impact the implementation of HCAP. This is due to the similarities in the interviewers’ training aspects and content between studies.

A key aspect of HCAP design is the inclusion of a computer assisted participant interview that can be implemented by a trained survey interviewer without the need for specialised individual such as a nurse or neuropsychologist. [12] It was clear that lay people can be as effective as nurses and research assistants, provided they receive rigorous training before being approved for interviewing and are provided on-going support and quality control review once in the field. The role of the interviewer is to ensure complete and accurate data collection, as well as establish rapport, build trust, and navigate sensitive topics related to cognitive function, aging, and health. Interviewers should be selected based on ability and competency. This requires robust training and accreditation procedures.

The interviewer’s role is nuanced and indispensable in ensuring the validity and depth of information gathered. Study data integrity relies on their ability to capture quality data and leave participants feeling comfortable about having participated. Interviewers should be made aware of just how important their role is to enhance their sense of importance in the wider team, building confidence and morale. The cognitive component of these particular interviews is a particular aspect that requires interviewers to be very comfortable in supporting participants to complete the HCAP protocol as much as possible without any coercive element, but in a way that does not interfere with standardisation of delivery. Ongoing quality control of interviewers and support to them is essential to pick up early variation in interviewers’ skills, as abandoning interviews can become an interviewers’ mode of dealing with discomfort at the content of an interview.

Fieldwork monitoring and quality control (QC) are essential components of any study and an important tool that improves data quality. [34] The ideal situation would be to include monitoring through the life course of the data collection phase (in real-time) including both the fieldwork team and the specialist researchers to allow for immediate corrective action if needed. Studies should review their system to ensure rigorous quality control is conducted in real-time and not just conducted on ad-hoc basis.

### Implementation and outputs

The three sub-themes within the framework are (1) Data collection, (2) Data preparation and (3) Study outcomes. Variation between studies depended on the local contexts (e.g., recruitment strategies, teams and organisational structure). It is important to maintain similar procedures for the administration of tests and data processing (cleaning and quality checks) to enable for robust pre-statistical harmonization. Good documentation and strong and immediate communication are essential to allow for any corrective actions to be implemented quickly. This includes communication of any adaptation with the wider network for feedback and transparency.

### Feedback and communication

From the interviews, PPI, focus group work and personal reflections of the researchers the themes related to this heading are (1) Feedback on protocol and study design (2) Feedback on study implementation and (3) Communication with the wider HCAP network. Feedback and communication help identify potential flaws or biases in the study design. It also helps gather perspectives from different members within the team to get the accurate picture about the challenges and barriers in the study implementation and how to improve them. Including Public and Participant Involvement (PPI) allows a better understanding of the diverse perspectives from older individuals that researchers may have not considered. Of significant importance is to keep good communication at all stages with HRS, the lead study, to ensure consistency and harmonization of data capture. HRS provides support for development of surveys using HCAP through technical assistance, staff training, and collaboration.

## Project limitations

This study had some challenges and limitations. Mainly, the comparison between studies was not straightforward, since the studies were administering different waves of HCAP and had dissimilar organisational structures and teams to implement the study. This sometimes meant that a question from the online questionnaire was understood differently across studies. However, we were still able to gather an in-depth understanding of each of the settings through the semi-structured interviews which clarified the answers to the questionnaire.

We were unable to pilot our questionnaire or our checklist for its utility and usability. However, the questionnaire was grounded in the research literature based on existing methods, theories and concepts. We also invite researchers to pilot our checklist. We have not reported on the differential impact of the data collection design or procedural differences of administration of cognitive measures on indicators of data quality (i.e. missing or incomplete data, and non-response) as this is beyond the scope of this paper. Although this is recognised part in the harmonization process. [22] Another factor that we were unable to investigate was funding, which would have been a critical determinant in implementation of HCAP across the four studies.

## Conclusion

Those conducting cross-study comparisons should give careful consideration to context and methods of data collection. [35] Investigators should take necessary steps to avoid selection and information bias to which all observational epidemiological studies are prone to and, if necessary, deliberate on whether any aspect of design bias should be considered in subsequent statistical analysis including weighting of survey data. This work highlights the importance of transparency, sharing of best practice and contributes towards HCAP goals of improving cross-national comparisons. [35] It is vital that researchers using these data are aware of the nature of fieldwork to better understand the variables and their context for more robust analyses.

Our framework and checklist, although not exhaustive, can be effectively applied for evaluating, planning, and implementing fieldwork for HCAP and tools for robust comparative research in measuring cognitive function among older adults. The framework provides a structured approach for identifying, mitigating, and monitoring sources of bias such as measurement error, confounding, and selection bias. We also include a summary of our recommendations for improving the implementation of HCAP in current and future studies (Box 2). Although designed for HCAP studies, the framework will be of value to other studies seeking to harmonize results or implement primary fieldwork across settings.

## Supplementary Material

Supplement 1

## Figures and Tables

**Table 1:** Description of the four English-speaking studies from HCAP network used in this study

Study	HCAP Wave under Investigation	Data Collection Format	Sample Selection with additional details
English Longitudinal Study of Ageing (ELSA)	Wave 2	Face to face interview with participant (respondent) in their residence. Family/friend (informant) completed questionnaire either in the respondent’s residence or could complete later and post. Also, option for a telephone interview.	Invited participants (n=3311) aged 65 and older who took part in ELSAS-HCAP Wave 1 and the most recent waves of the core study, Wave 9 (2018/2019) and/or Wave 10 (2023/2024) ELSA-HCAP Wave was 1 completed in 2018 with 1273 participants (82% of sample had informant interview) [14]*
Health and Retirement Study (HRS)	Wave 2	Face to face interview for both participant (respondent) and family/friend (informant) took place in the respondent’s residence.	Invited participants (n=4126) aged 65 and older were those who took part in HRS-HCAP Wave 1 and took part in 2022/2023 of core HRS study. HRS-HCAP Wave 1 was completed in 2016 with 3,496 participants (87% of sample with informant interview) [12]*
Northern Ireland Cohort for the Longitudinal Study of Ageing (NICOLA)	Wave 1	Face to face interview with participant (respondent) in their residence or at research centre Informant interview: Face to face interview following participant interview (i.e. during the same visit) OR telephone interview at a later date. Also had option to complete questions online.	Invited participants (n=2077) aged 65 and older taking part in (Wave 2, 2017–2022) of core NICOLA study.
The Irish Longitudinal Study on Ageing (TILDA)	Wave 1	Face to face interview with (respondent) participant in their residence Informant interview: Telephone interview	Invited participants (n=1830) aged 65 and older taking part in the most recent wave from core TILDA (Wave 6, 2021)

Abbreviations: HCAP; Harmonized Cognitive Assessment Protocol.

* References

**Table 2:** Summary of data collection time periods and the average number of participants interviewed per month across each of the four studies, NICOLA, TILDA, HRS and ELSA.

Study	Start and End Dates of Data Collection	Length of Fieldwork (months)	Interviewer Type	Number of Interviewers	Final number in cohort	Average (mean) interviews/month	Average (mean) / interviewer
ELSA^a^	24^th^ Apr to 31^st^ Oct 2023	6.2 months	Lay interviewer	88	2022	326	23
HRS^a^	20^th^ July 2022 to 9^th^ Dec 2023	16.3 months	Lay interviewer	49	4126	253	84
NICOLA^a^	7^th^ Feb 2022 to 4^th^ Dec 2023	22 months	RA/nurse	6	1037	47	172
TILDA^a^	6th Dec 2021 to 15th Dec 2023	24 months	RA/nurse	6	1344	56	224

a Abbreviations: ELSA, English Longitudinal Study of Ageing; HRS, Health and Retirement Study, NICOLA, Northern Ireland Cohort for the Longitudinal Study of Ageing; RA, Research Assistant; TILDA, The Irish Longitudinal Study on Ageing

## Box 2: Recommendations for the effective implementation and HCAP for future studies

### Integrated working:

Develop systems for greater integrated working between the fieldwork team collecting data and the specialist research team responsible for coordinating and analysing the data. This is particularly relevant when the organisational model is not all in-house or under one organisation. Fast and timely communication and easier accessibility to the raw data improves QC checks, mitigate challenges and allows for faster corrective actions. Oversight of the study helps bridge the gap between those leading and coordinating HCAP and the fieldwork team.

### Accreditation and training:

Quality, length, and outcome of training are key to standardising the method of data collection and reducing the interviewers’ judgement.

### Fieldwork team:

Larger interviewer numbers speed up data collection phase and allow greater geographical coverage but can be at the expense of increasing variability and interviewer bias. Managing a larger fieldwork team requires effective management and coordination for timely communication between the fieldworkers and the specialist researchers to identify issues and provide real-time solutions.

Providing structural support from the specialist researchers to the interviewers during fieldwork and emphasising the importance of the interviewer’s role during the training is essential.

### Fieldwork feedback:

Having regular meetings with interviewers (when less than 10 are involved in the data collection) or feedback through focus groups (more than 10) during fieldwork implementation allow the specialist researchers to learn about the challenges encountered in the field and implement real-time solutions.

### Quality checks:

It is important to maximise on real-time data checks and feedback to the interviewers to correct and prevent systematic errors.

### Public and Participant Involvement (PPI):

Including a PPI component is recommended for HCAP and similar studies. This can provide insights into further adaptation of HCAP to suit the local context. PPI requires sufficient resources with study information presented in a format that is clear and easy to understand. Researchers must be mindful of ‘power dynamics’ to ensure PPI members feel confident in presenting ideas and suggestions.
