# Supplementary material for: A study evaluation framework for measuring cognition. Lessons learned in cross-national contexts from four English-speaking aging cohorts
Source: Res Sq. 2024 Dec 20:rs.3.rs-5574616. Preprint. [Version 1] doi: 10.21203/rs.3.rs-5574616/v1 (PMC11702860; doi:10.21203/rs.3.rs-5574616/v1)
Supplement: Supplement 1 [file NIHPPRS5574616v1-supplement-1.pdf]

Supplementary Files

This is a list of supplementary files associated with this preprint. Click to download.

- SupplementaryInformationevaluation.pdf
